# Assessment of tumor hypoxia and perfusion in recurrent glioblastoma following bevacizumab failure using MRI and ^18^F-FMISO PET

**DOI:** 10.1038/s41598-021-84331-5

**Published:** 2021-04-07

**Authors:** Shiliang Huang, Joel E. Michalek, David A. Reardon, Patrick Y. Wen, John R. Floyd, Peter T. Fox, Geoffrey D. Clarke, Paul A. Jerabek, Kathleen M. Schmainda, Mark Muzi, Hyewon Hyun, Eudocia Quant Lee, Andrew J. Brenner

**Affiliations:** 1 Mays Cancer Center, The University of Texas Health Science Center at San Antonio, 7703 Floyd Curl Drive, San Antonio, TX 78229-3900 USA; 2 Dana-Farber Cancer Institute, Boston, MA USA; 3 Departments of Radiology and Biophysics, Medical College of Wisconsin, Wauwatosa, WI USA; 4 Department of Radiology, University of Washington, Seattle, WA USA; 5 Division of Nuclear Medicine, Department of Radiology, Brigham and Women’s Hospital, Boston, MA USA

**Keywords:** Cancer imaging, CNS cancer, Cancer microenvironment

## Abstract

Tumoral hypoxia correlates with worse outcomes in glioblastoma (GBM). While bevacizumab is routinely used to treat recurrent GBM, it may exacerbate hypoxia. Evofosfamide is a hypoxia-targeting prodrug being tested for recurrent GBM. To characterize resistance to bevacizumab and identify those with recurrent GBM who may benefit from evofosfamide, we ascertained MRI features and hypoxia in patients with GBM progression receiving both agents. Thirty-three patients with recurrent GBM refractory to bevacizumab were enrolled. Patients underwent MR and ^18^F-FMISO PET imaging at baseline and 28 days. Tumor volumes were determined, MRI and ^18^F-FMISO PET-derived parameters calculated, and Spearman correlations between parameters assessed. Progression-free survival decreased significantly with hypoxic volume [hazard ratio (HR) = 1.67, 95% confidence interval (CI) 1.14 to 2.46, P = 0.009] and increased significantly with time to the maximum value of the residue (Tmax) (HR = 0.54, 95% CI 0.34 to 0.88, P = 0.01). Overall survival decreased significantly with hypoxic volume (HR = 1.71, 95% CI 1.12 to 12.61, p = 0.01), standardized relative cerebral blood volume (srCBV) (HR = 1.61, 95% CI 1.09 to 2.38, p = 0.02), and increased significantly with Tmax (HR = 0.31, 95% CI 0.15 to 0.62, p < 0.001). Decreases in hypoxic volume correlated with longer overall and progression-free survival, and increases correlated with shorter overall and progression-free survival. Hypoxic volume and volume ratio were positively correlated (r_s_ = 0.77, P < 0.0001), as were hypoxia volume and T1 enhancing tumor volume (r_s_ = 0.75, P < 0.0001). Hypoxia is a key biomarker in patients with bevacizumab-refractory GBM. Hypoxia and srCBV were inversely correlated with patient outcomes. These radiographic features may be useful in evaluating treatment and guiding treatment considerations.

## Introduction

Glioblastoma (GBM) is the most common and most aggressive of primary malignant brain tumors in adults, with an annual incidence of 3.19 per 100,000 and 2-year and 5-year survival rates of 25% and 5% to 10%, respectively^1^. Standard treatment options include radiation, chemotherapy with temozolomide, and tumor-treating fields in the newly diagnosed setting^2,3^ followed by a bevacizumab (Bev) containing regimen upon progression. Thereafter, no subsequent therapeutic has delayed progression or improved survival, with a median progression-free survival (PFS) of 1.6 months and median overall survival (OS) of 4 months for patients without bevacizumab (Bev) treatment^4^.

Hypoxia is the reduction of oxygen to tissues below physiologic levels (i.e., 10 mmHg, 2% O_2_ in tumors) and is generally caused by an inadequate blood supply. The ensuing imbalance between oxygen delivery and oxygen consumption can render tumor cells hypoxic and thus more resistant to anticancer therapy such as radiation^5^. Therefore, factors that regulate the hypoxic state represent potential targets for treatment.

Given the resulting increased tumor aggressiveness, metastatic spread, resistance to therapy, rate of recurrence, and decreased local control and survival^6,7^, hypoxia measurements could improve treatment planning and early assessment of efficacy in GBM. An imaging method is the most desirable approach for measuring hypoxia because it is noninvasive, can provide high spatial resolution, has reasonable cost, and is easy to use in clinical trials^8^. ^18^F-Fluoromisonidazole (^18^F-FMISO) PET is currently the most widely studied PET imaging method to determine tissue hypoxia^6,9^. In an oxygen-depleted environment, ^18^F-FMISO is retained in viable hypoxic cells^10^ and is related to the severity of hypoxia^11,12^.

Another imaging modality, MRI, can reveal pathophysiologic processes associated with GBM. The current radiologic standard in evaluating response is the Response Assessment in Neuro-Oncology (RANO) criterion, based on 2-dimensional measurement of conventional T1 enhancing and fluid-attenuated inversion recovery (FLAIR) MR images. Conventional MRI only provides anatomic, not pathology-specific, information^13^. Thus, advanced quantitative imaging techniques are being explored. Dynamic susceptibility contrast MRI (DSC-MRI) is the most prevalent method for measuring brain tumor perfusion^14^. It is a bolus tracking technique that rapidly acquires gradient echo or spin echo images before, during, and after first-pass transit through the brain of an exogenous paramagnetic gadolinium-based contrast agent that transiently decreases MR signal intensity^15^. Parameters derived from DSC-MRI include normalized regional cerebral blood volume (nrCBV)^16,17^, standardized rCBV (srCBV)^16,17^, normalized regional cerebral blood flow (nrCBF)^18^, mean transit time (MTT)^19^, time to peak (TTP)^19^, and the time to the maximum value of the residue function (Tmax)^19^. One or more of these parameters are reliable biomarkers for grading tumors, predicting malignant transformation, planning treatments, and monitoring responses^14^.

As part of a multicenter, phase 2 trial of the hypoxia-targeting prodrug evofosfamide^20^ (Evo) plus Bev (TH-302, NCT02342379^21^), we explored the extent of tumor hypoxia in vivo via ^18^F-FMISO PET imaging and evaluated DSC-MRI perfusion parameters in patients with Bev-refractory recurrent GBM.

## Patients and methods

Seventeen patients were recruited from UT Health San Antonio and 16 from Dana-Farber Cancer Institute. The inclusion criteria included adults with (a) ECOG (Eastern Cooperative Oncology Group) 2 or less and histologically confirmed GBM, and (b) progression determined by RANO criteria after standard combined radiation and temozolomide chemotherapy, plus Bev. All patients had received Bev at 10 mg/kg intravenously (IV) every 2 weeks and Evo at 670 mg/m^2^ IV every 2 weeks, in 6-week cycles, until disease progression. Patients underwent baseline assessment for hypoxic burden by ^18^F-FMISO PET, DSC imaging, and serum sampling for biomarker analysis. Baseline and 28-day MRI and PET imaging were acquired within 3 days prior to treatment and every 4 weeks after starting treatment. Patients were excluded from the study if their PET and MRI data were not sufficient for further analysis. The protocol was approved by the Institutional Review Boards at UT Health San Antonio and Dana Farber Cancer Institute, all patients provided informed consent before enrollment in the study, and all methods were carried out in accordance with Good Clinical Practice and local guidelines and regulations.

### Image acquisition

MRI scans were performed on 3 T MRI scanners (Philips, GE, or Siemens). Each scanning session consisted of 3D pre- and post-contrast T1 weighted, FLAIR, diffusion-weighted MRI (DWI), dynamic contrast enhanced (DCE), and DSC MR images. T1 pre-contrast and FLAIR images were acquired before contrast injection. DCE-MRI and DWI were acquired after the first intravenous injection of 0.1 mmol/kg of a standard gadolinium-based contrast agent. For DCE-MRI, injection took place after 10 baseline frames were obtained. The second injection was for DSC-MRI and T1 post contrast images.

FLAIR images were acquired with TR = 10000 ms, TE = 100 ms, time of inversion (TI) = 2500 ms, and matrix = 270 × 320, slice = 52. 3D T1 weighted images were acquired using gradient echo. TR = 2100 ms, TE = 5 ms, and matrix size = 256 × 256 × 192. DSC-MRI images using gradient-echo (GRE) (17 patients) or spin-echo (SE) echo planar images (16 patients) were acquired with the following recommended parameters: GRE TR = 1500 ms, TE = 30 ms, SE TR = 2000 ms, TE = 60–150, flip angle = 70–90^22^, slice thickness = 3–5 mm, matrix size = 128 × 128 and time point is 60–120.

PET scans were performed on two devices, both of which were calibrated. On a CTI EXACT HR + scanner (Knoxville, TN), acquisition parameters were 63 slices; 2.4-mm thickness; and image size: 128 × 128 × 63. The images were reconstructed using 3D iterative reconstruction with four iterations, 16 subsets, zoom = 2; Gaussian kernel FWHM 5.0 mm, 2D measured attenuation correction, axial filtering, and scatter correction). On a Siemens Biograph40 mCT scanner, acquisition parameters were 75 slices; 3-mm thickness; and image size: 128 × 128 × 75. The images were reconstructed using 3D iterative ordered-subset expectation maximization with two iterations and 21 subsets, time of flight, point-spread function-correction, slice thickness 3 mm, matrix size 128 × 128, in-plane reconstruction pixels size 6.3638 mm × 6.3638 mm, and a Gaussian post-reconstruction convolution kernel with full width at half maximum of 5 mm. A CT scan was used for attenuation correction.

In all cases, patients were injected intravenously with 3.7 MBq/kg of ^18^F-FMISO. A 20-min static ^18^F-FMISO PET emission image was acquired at about 120 min after injection of ^18^F-FMISO.

### Survival analyses

Overall survival time was counted from enrollment until death or end of follow-up. Progression time was counted from enrollment until progression or death or end of follow-up. The enrollment date was used as start date, and either the death date or last follow-up date was used as the endpoint. Patients who were lost to follow-up or survived to the end of follow-up were considered censored.

### Image analyses

All imaging data were post-processed using MATLAB2016 (Math Works) and OsiriX (Pixmeo, Geneva, Switzerland) with the IB Rad Tech plugin (Imaging Biometrics LLC, Elm Grove, WI). Briefly, using the IB Rad Tech tool, the following processing steps were performed with minimal user intervention. First, T1-pre contrast images were co-registered to T1-post contrast images. Next, delta T1 images were generated based on differences between the standardized T1-post contrast and standardized T1-pre contrast images^23^. Likewise, the DSC images were registered to T1-post contrast images. IB Rad Tech then directs the user to manually draw a reference region of interest (ROI) in normal-appearing white matter to create normalized rCBV and CBF maps (nrCBV, nrCBF). In addition, using the registered DSC-MRI data, IB Rad Tech computed srCBV^22^, rCBF, MTT, TTP and Tmax maps, which did not require a reference ROI.

Tumor ROIs were manually drawn on the T1-post contrast images which, according to convention, include the radiologic necrotic region. The total T1 weighted tumor volume was calculated. In addition, an empirically determined threshold of 3000 (IB Rad Tech plugin) was applied to the delta T1 maps within the tumor ROIs to extract the enhancing tumor ROIs (without necrosis) and the subset ROIs that include radiological necrosis only. The enhancing and radiologic necrotic tumor volumes (T1_Vol_et and T1_Vol_nt) were calculated.

The FLAIR ROIs, which typically include both the tumoral and peritumoral regions, were manually drawn on the FLAIR images. The FLAIR tumor volume, FLAIRΔT1 (FLAIR_Vol excluded T1_Vol) and the Volume ratio (T1_Vol/ FLAIR_Vol) were calculated. These ROIs were then applied to the different perfusion parameter maps. The mean, median, and the maximal value of the MTT, TTP, nrCBV, srCBV, nrCBF and Tmax within tumor ROIs were calculated.

FLAIR images were co-registered to the ^18^F-FMISO PET images and the FLAIR tumor ROIs were used to determine the tumor ROIs on the ^18^F-FMISO PET images. Because FMISO is a freely diffusing tracer, the tumor ROIs on the ^18^F-FMISO PET images were determined by expanding the FLAIR ROIs to include all regions where there was FMISO uptake, which extended beyond the original FLAIR tumor ROI^24^. Two 2 cm diameter ROIs on both sides of the cerebellar cortex were used as the image derived blood surrogate to determine the surrogate of tissue to blood ratio (TB ratio)^9^, the mean value of hypoxia volume (HVmean) and the mean of the top 5% of TB pixels (TB5percent). HV was determined by the number of pixels with values above 1.2 on the TB image^9^. Other parameters, such as SUVmax, SUVpeak, TBmax, and TBpeak, were determined within the tumor ROIs.

### Statistical methods

OS and PFS were graphically described with Kaplan–Meier curves. Imaging parameters were summarized in original units with the median, minimum, and maximum. The significance of variations in OS and PFS with PET and MRI brain imaging parameters were assessed with univariate and multivariate proportional hazards models in log units and standardized to mean zero and unit variance. Results were summarized as hazard ratios (HR) and 95% confidence intervals (CI). The significance of variation in imaging parameters, OS, and PFS with clinic site was assessed with principal component analysis (PCA) and univariate and multivariate proportional hazards models in log units. Multivariate proportional hazards models were fit on the first two principal components, clinic site, and interactions with clinic site. Imaging data were stratified in original units at the median and the resulting survival distributions in each stratum were described with Kaplan–Meier curves and compared with log-rank tests. Relationships between OS and PFS and all log-transformed and standardized imaging parameters were assessed with proportional hazards models and stepwise forward selection; the resulting reduced models were described with stratification, stratum-specific Kaplan–Meier curves and log-rank tests. Spearman correlations between imaging parameters in original units were graphically described with the corrplot R package and the hclust option. SAS Version 9.4 for Windows (SAS Institute, Cary, North Carolina) and R were used throughout (Supplementry Information).

### Previous presentation

Some of the data in this article were previously presented at the 2016 Annual Meeting of the American Society for Clinical Oncology, the 22nd annual meeting of the Society for Neuro-Oncology (SNO), San Francisco, California, in 2017 and the SNO 23rd annual meeting, Louisiana, New Orleans, in 2018.

## Results

Forty-one subjects were screened for this study; 6 were screen failures (Dana Farber Elevated LFT 1, Declining performance status 1, UT Health Declining performance status 3, Thrombocytopenia and anxiety 1), 35 were enrolled (Dana Farber 17, UT Health 18) and two withdrew consent (Dana Farber 1, UT Health 1). Of the remaining 33, the mean patient age was 46 years (range, 19–76 years). 22 were male and 11 were female, and the ECOG performance status was 0 or 1 in 82.9% of patients. All of the 33 enrolled patients failed standard treatment (randomized in pre-surgery cohorts 1–3 with 9 proceeding to Evo/Bev after surgery and the remainder proceeding directly to Evo/Bev). Of the 33, a total of 28 (84.5%) progressed [Dana Farber 14 (87.5%), UT Health 14 (82.4%)] and 30 (90.9%) died [Dana Farber 15 (93.8%), UT Health 15 (88.2%)]. Of the first 11 patients, 2 were progression-free at 4 months (120 days), allowing the trial to proceed to the second stage. Of the 33, 8 [Dana Farber 4 (25%), UT Health 4 (23.5%)] were progression-free at 4 months. The demographic data (age, race, gender) of all included patients are shown in Table 1.

**Table 1:** The demographic data (age, race, gender) of all patients.

Demographic	Dan Farber (n = 16)	UT Health (n = 17)	Total (n = 33)	P-value
Age mean ± SD	45.2 ± 14.6	47.3 ± 16.92	46.3 ± 15.66	0.71
Male n (%)	9 (56.3)	13 (76.5)	22 (66.7)	0.22
**Race n (%)**				
White	13 (81.3)	17 (100)	30 (90.9)	0.1
Black or African American	1 (6.3)	0 (0)	1 (3)	
Other	2 (12.5)	0 (0)	2 (6.1)	

**Figure 1:**
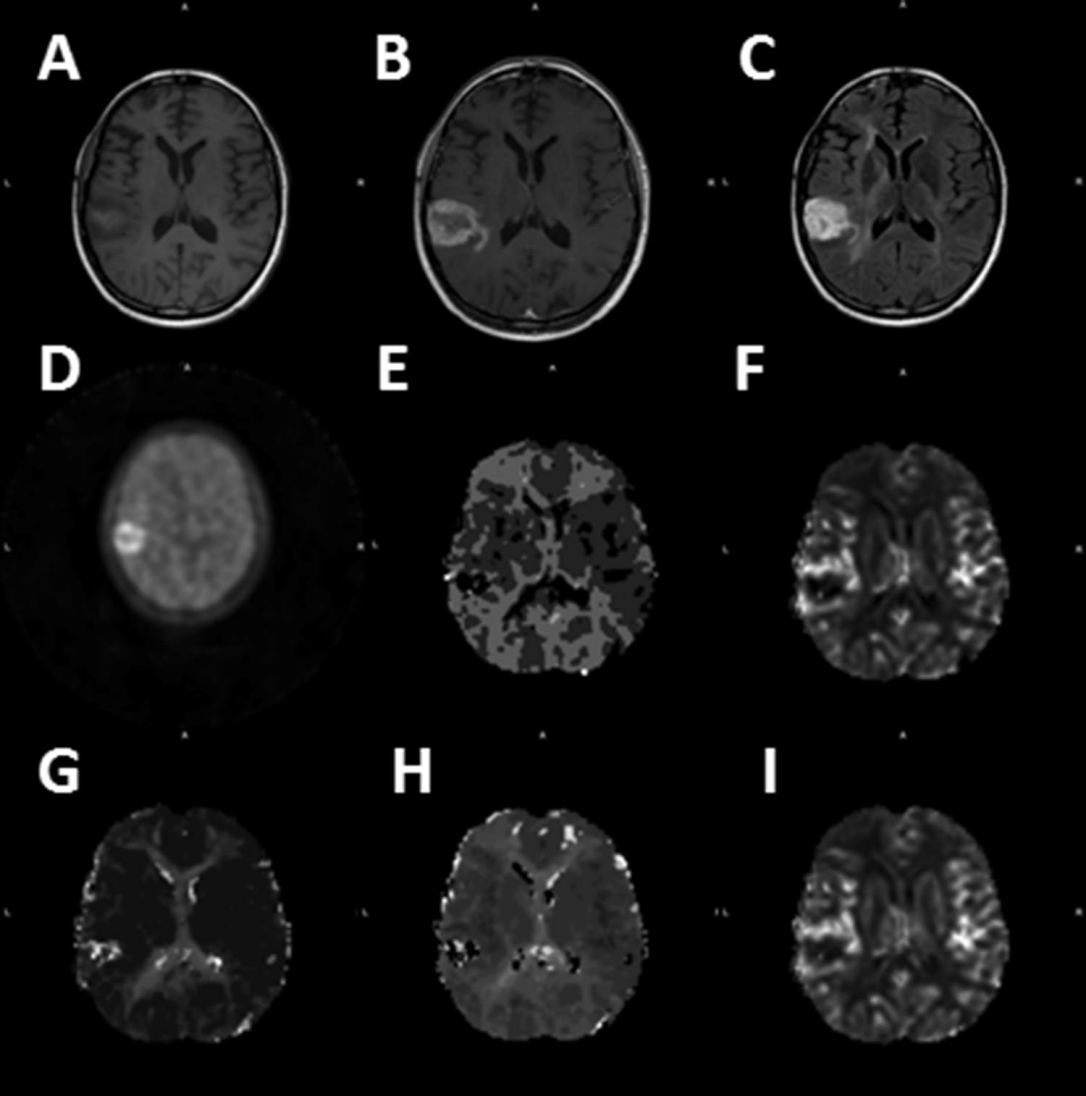
Representative images at baseline (Case UT003). (**A)** pre-contrast T1, (**B)** post-contrast T1, (**C)** FLAIR, (**D)** ^18^F-FMISO PET, (**E)** MTT, (**F)** srCBV, (**G)** Tmax, (**H)** TTP, (**I)** nrCBF.

Figure 1 shows an example from Patient UT003 at baseline for pre-contrast T1-weighted image (Fig. 1A), post-contrast T1-weighted image (Fig. 1B), FLAIR image (Fig. 1C) and ^18^F-FMISO PET image (Fig. 1D). Representative corresponding MTT (Fig. 1E), srCBV (Fig. 1F), Tmax (Fig. 1G), TTP (Fig. 1H) and nrCBF (Fig. 1I) maps are shown as well.

**Figure 2:**
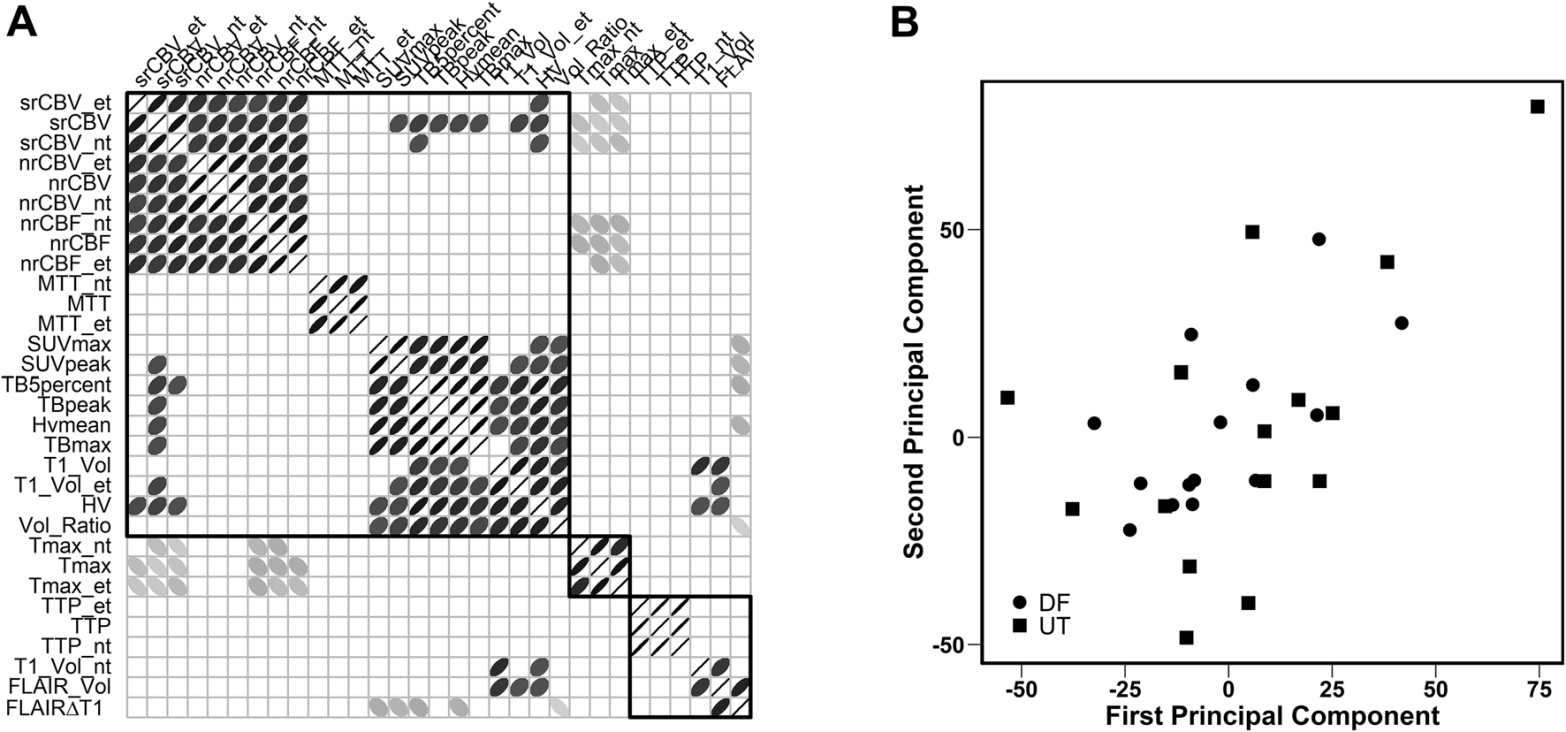
** (A)** Spearman correlations between all imaging parameters. Significant correlations (p ≤ 0.05) are indicated with ellipses with the shape of each ellipse reflecting the direction and strength of the correlation. Positive correlations are described with ellipses directed from the lower left to the upper right and negative correlations are described with ellipses directed from the upper left to the lower right. Strong correlations are described with thin ellipses and weak correlations with thick ellipses. Non-significant correlations are indicted with blanks. (**B)** Scatter plot of the first and second principal components by clinic based all imaging parameters in n = 33 subjects; the centroids were not significantly different (p = 0.73).

We extracted the mean imaging parameters within the different ROIs. The median, minimum, and maximum of these mean parameters at baseline are shown in Table 2. The median enhancing tumor volume was 33.2 cm^3^ (range 0.7–185 cm^3^). The median T2 FLAIR tumor volume was 101.9 cm^3^ (range 17.9–275.1 cm^3^). The median HV was 28.5 cm^3^ (range 0–155.1 cm^3^). The median srCBV within T1_Vol ROI was 1.2 (range 0.5–5.1). The associations between imaging parameters among the 33 patients are shown in Fig. 2A.

**Table 2:** The Median, Min and Max of imaging parameters on baseline imaging (n = 33).

Category	Parameter	Median	Minimum	Maximum
Anatomical	FLAIR_Vol	101.9	17.9	275.1
	FLAIRΔT1	50.5	6.6	188.1
	T1_Vol	33.2	0.7	185
	T1_Vol_et	19.1	0.1	102.1
	T1_Vol_nt	13.1	0.5	115.2
	Vol_Ratio	0.4	0	0.8
Perfusion	MTT	4.5	2.7	28.7
	MTT_et	4.5	2.7	36.9
	MTT_nt	4.1	2.7	20.8
	Tmax	4.1	1.3	14.1
	Tmax_et	3.6	1.2	14.1
	Tmax_nt	4.7	1.4	14.4
	nrCBF	1.3	0	5.7
	nrCBF_et	1.5	0	6
	nrCBF_nt	1.3	0	4.1
	nrCBV	1.5	0.6	106.8
	nrCBV_et	1.5	0.5	133.2
	nrCBV_nt	1.4	0.5	82
	srCBV	1.2	0.5	5.1
	srCBV_et	1.4	0.5	5.3
	srCBV_nt	1.1	0.5	3.2
PET	HV	28.5	0	155.1
	Hvmean	1.3	1.2	1.9
	SUVmax	2.6	1.3	5.8
	SUVpeak	2.1	1	5.2
	TB5percent	1.4	1.1	2.9
	TBmax	1.9	1.2	3.3
	TBpeak	1.6	1.1	3.1

*T1_Vol*: Tumor volume on post T1 weighted image, *T1_Vol_et*: enhancing tumor volume, *T1_Vol_nt*: necrotic tumor tissue volume on post T1 weighted image, *srCBV*: the mean value within T1_Vol, *srCBV_et*: the mean value within T1_Vol_et, *srCBV_nt*: the mean value within T1_Vol_nt, *FLAIRΔT1*: FLAIR_Vol - T1_Vol, *Vol_Ratio*: T1_Vol/ FLAIR_Vol.

Significant Spearman correlations were found between HV and T1_Vol (r_s_ = 0.75, P < 0.001), HV and ratio of post contrast T1to FLAIR tumor volume (Vol_Ratio) (r_s_ = 0.69, P < 0.001), HV and T1_Vol_et (r_s_ = 0.71, P < 0.001), TB5percent and T1_Vol_et (r_s_ = 0.64, P < 0.001), and TB5percent and Vol_Ratio (r_s_ = 0.67, P < 0.001).

Moderate positive Spearman correlations were found between TB5percent and srCBV (r_s_ = 0.52, P = 0.004), HV and srCBV (r_s_ = 0.50, P = 0.006), TBpeak and srCBV (r_s_ = 0.46, P = 0.01), TB5percent and T1_Vol (r_s_ = 0.55, P < 0.001), TBpeak and Vol_Ratio (r_s_ = 0.59, P < 0.001), TBpeak and T1_Vol (r_s_ = 0.46, P = 0.009), TBpeak and T1_Vol_et (r_s_ = 0.57, P < 0.001), HVmean and Vol_Ratio (r_s_ = 0.58, P < 0.001), TBmax and T1_Vol_et (r_s_ = 0.46, P = 0.008).

**Figure 3:**
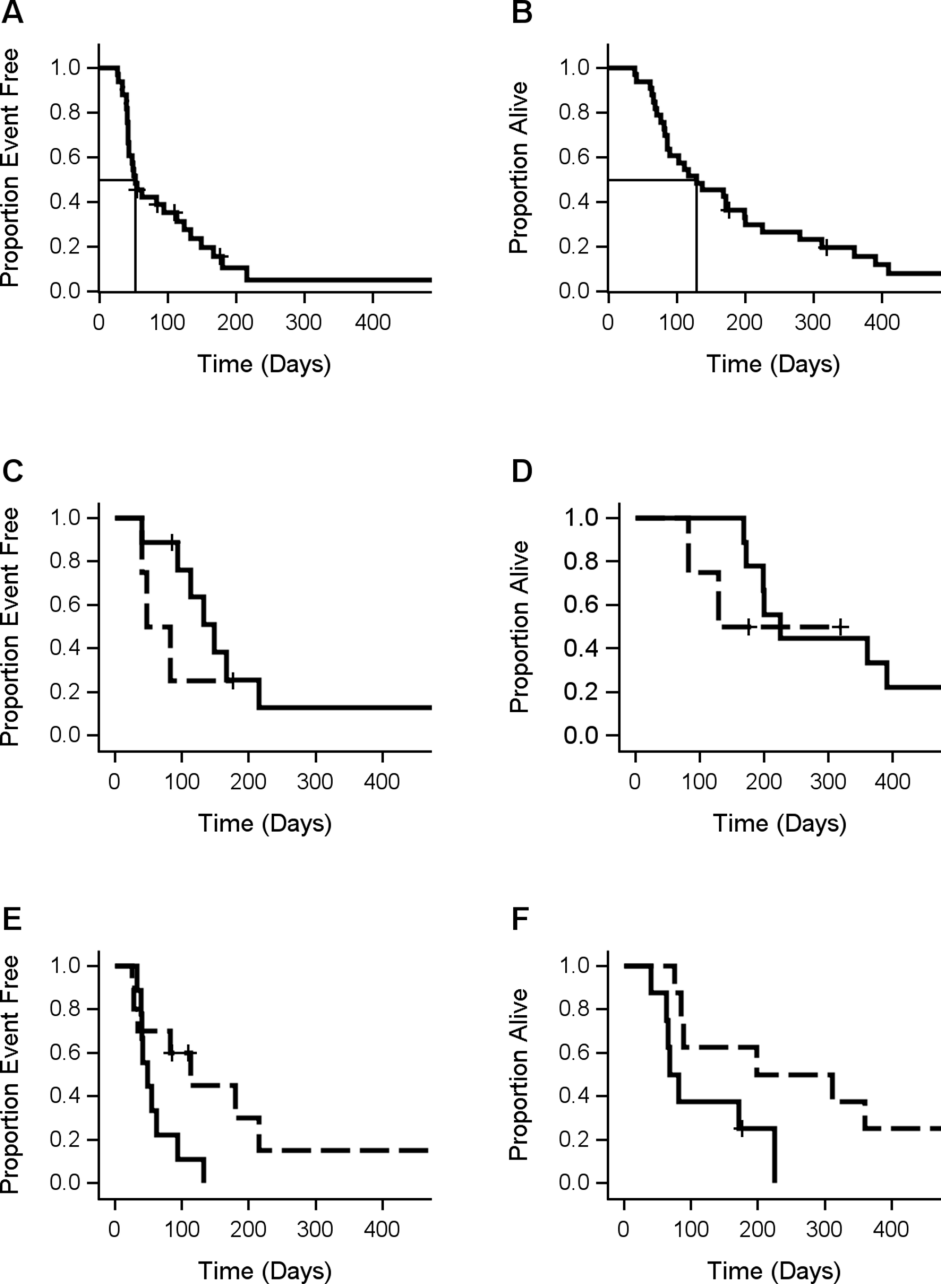
** (A)** Progression-free survival in n = 33 patients. (**B)** Overall survival in n = 33 patients. (**C)** Progression-free survival in n = 13 patients with repeated imaging data stratified by HV Change (= HV at follow-up minus HV at baseline) < 0 (Solid, n = 9) and HV Change ≥ 0 (Dashed, n = 4); p = 0.42, (**D)** Overall survival in n = 13 patients with repeated imaging data stratified by HV Change (= HV at follow-up minus HV at baseline) < 0 (Solid, n = 9) and HV Change ≥ 0 (Dashed, n = 4); p = 0.55, (**E)** Progression-Free Survival in n = 19 patients with stratified by Tmax < 4.05 and HV ≥ 28.52 (Solid, n = 9) and Tmax ≥ 4.05 and HV < 28.52 (Dashed, n = 4) (p = 0.05) based on forward selection from a multivariate model of progression-free survival in terms of all imaging parameters, (**F)** Overall survival in n = 16 patients with stratified by nrCVB_et ≥ 1.5 and Tmax < 4.05 (Solid, n = 8) and nrCBV_et < 1.5 and Tmax ≥ 4.05 (Dashed, n = 8) (p = 0.04) based on forward selection from a multivariate model of overall survival in terms of all imaging parameters.

The median times to progression and death were 53 days (95% CI 42 to 113) and 129 days (95% CI 86 to 199 days), respectively (Fig. 3A,B).

The first two principal components, denoted as PCA1 and PCA2, of all imaging biomarkers in 33 patients are summarized in Fig. 2B by clinic site. The mean of the first two principal components did not vary significantly with clinic site (Dana Farber PCA1 − 2.2 ± 20.1, PCA2 1.9 ± 20.2, UT Health PCA1 4.5 ± 30.7, PCA2 2.5 ± 34.5, P = 0.73). In proportional hazards modeling of OS, clinic site and all interactions were non-significant (P = 0.34) and thus were removed from the model. Subsequently, clinic site did not contribute significantly to the model and was removed. In the resulting model, PCA1 and PCA2 did not contribute significantly (PCA1 HR = 1.01, 95% CI 0.986 to 1.034, P = 0.41, PCA2 HR = 1.016, 95% CI 0.992 to 1.040, P = 0.20). After removing PCA2, PCA1 contributed significantly to the results (HR = 1.021, 95% CI 1.003 to 1.041, P = 0.03).

In a proportional hazards model of PFS in terms of clinic site, PCA1 and PCA2, and pairwise interactions, all interactions were non-significant and were removed from the model. In the resulting main effects model, clinic site did not contribute significantly (P = 0.32) and was removed; in the reduced model PCA1 and PCA2 did not contribute significantly (PCA1 HR = 1.019, 95% CI 0.994 to 1.044, P = 0.15, PCA2 HR = 1.002, 95% CI 0.982 to 1.023, P = 0.84). After removing PCA2, PCA1 contributed significantly to the results (HR = 1.020, 95% CI 1.002 to 1.039, P = 0.03).

Results of univariate propoprtional hazards modeling of PFS and OS with brain imaging parameters are summarized in Table 3. PFS increased significantly with Tmax (HR = 0.54, 95% CI 0.34 to 0.88, P = 0.01), Tmax_nt (HR = 0.55, 95% CI 0.33 to 0.9, P = 0.02), and Tmax_et (HR = 0.57, 95% CI 0.35 to 0.94, P = 0.03) and decreased significantly with HV (HR = 1.67, 95% CI 1.14 to 2.46, P = 0.009) in univariate PH models.

**Table 3:** Univariate proportional hazards models of PFS and OS.

Category	Parameter	Progression free survival			Overall survival		
		HR	95% CI	p-value	HR	95% CI	p-value
Anatomical	FLAIR_Vol	1.32	(0.91, 1.92)	0.15	1.53	(1, 2.36)	0.05
	FLAIR_edma	1.18	(0.78, 1.79)	0.44	1.18	(0.77, 1.8)	0.45
	T1_Vol	1.43	(0.95, 2.13)	0.08	1.85	(1.17, 2.92)	0.009
	T1_Vol_et	1.26	(0.89, 1.79)	0.19	1.5	(1.02, 2.22)	0.04
	T1_Vol_nt	1.44	(0.91, 2.3)	0.12	1.77	(1.05, 3)	0.03
	Vol_Ratio	1.35	(0.9, 2.03)	0.14	1.4	(0.92, 2.14)	0.12
Perfusion	MTT	0.77	(0.36, 1.66)	0.51	0.98	(0.57, 1.7)	0.95
	MTT_et	0.76	(0.36, 1.6)	0.47	0.98	(0.62, 1.55)	0.94
	MTT_nt	0.7	(0.2, 2.5)	0.58	0.98	(0.3, 3.24)	0.98
	Tmax	0.54	(0.34, 0.88)	0.01	0.31	(0.15, 0.62)	< 0.001
	Tmax_et	0.57	(0.35, 0.94)	0.03	0.31	(0.14, 0.65)	0.002
	Tmax_nt	0.55	(0.33, 0.9)	0.02	0.41	(0.22, 0.74)	0.003
	nrCBF	1.04	(0.68, 1.6)	0.84	1.24	(0.74, 2.1)	0.42
	nrCBF_et	1.05	(0.7, 1.58)	0.8	1.26	(0.78, 2.03)	0.34
	nrCBF_nt	1.01	(0.69, 1.49)	0.95	1.08	(0.66, 1.76)	0.76
	nrCBV	0.5	(0.05, 5.08)	0.56	1.1	(0.77, 1.57)	0.61
	nrCBV_et	0.49	(0.04, 5.96)	0.57	1.1	(0.77, 1.57)	0.61
	nrCBV_nt	0.48	(0.04, 6.3)	0.58	1.09	(0.76, 1.57)	0.62
	srCBV	1.2	(0.84, 1.71)	0.32	1.61	(1.09, 2.38)	0.02
	srCBV_et	1.16	(0.82, 1.65)	0.4	1.56	(1.07, 2.26)	0.02
	srCBV_nt	1.17	(0.84, 1.64)	0.35	1.46	(1, 2.14)	0.05
PET	HV	1.67	(1.14, 2.46)	0.009	1.71	(1.12, 2.61)	0.01
	Hvmean	1.21	(0.89, 1.65)	0.23	1.22	(0.85, 1.75)	0.28
	SUVmax	1.12	(0.83, 1.52)	0.46	1.21	(0.84, 1.75)	0.3
	SUVpeak	1.14	(0.85, 1.53)	0.38	1.24	(0.86, 1.79)	0.25
	TB5percent	1.26	(0.92, 1.72)	0.16	1.29	(0.89, 1.88)	0.17
	TBmax	1.23	(0.89, 1.7)	0.21	1.13	(0.81, 1.57)	0.48
	TBpeak	1.26	(0.91, 1.73)	0.17	1.26	(0.87, 1.83)	0.22

OS increased significantly with Tmax (HR = 0.31, 95% CI 0.15 to 0.62, P < 0.001), Tmax_nt (HR = 0.41, 95% CI 0.22 to 0.74, P = 0.003), Tmax_et (HR = 0.31, 95% CI 0.14 to 0.65, P = 0.002) and decreased significantly with HV (HR = 1.71, 95% CI 1.12 to 2.61, P = 0.01) and the anatomic MRI parameters T1_Vol (HR = 1.85, 95% CI 1.17 to 2.92, P = 0.009), T1_Vol_nt (HR = 1.77, 95% CI 1.05 to 3, P = 0.03), T1_Vol_et (HR = 1.5, 95% CI 1.02 to 2.22, P = 0.04), FLAIR_Vol (HR = 1.53, 95% CI 1 to 2.36, P = 0.05), srCBV (HR = 1.61, 95% CI 1.09 to 2.38, P = 0.02), srCBV_nt (HR = 1.46, 95% CI 1 to 2.14, P = 0.05), and srCBV_et (HR = 1.56, 95% CI 1.07 to 2.26, P = 0.02). Clinic site was not included in the models summarized in Table 3.

Thirteen patients underwent MRI and PET imaging at baseline and 4 weeks after treatment. We separated patients into two groups, detailed in the figure legends, based on positive versus negative HV change. In general, the negative group experienced a longer OS and PFS and the positive group a shorter OS and PFS (Figs. 3C,D).

### Multivariate analysis of PFS and OS

After forward selection, the final PFS model contained only Tmax and HV (Tmax HR = 0.54, P = 0.01, HV HR = 1.6, P = 0.046). The joint distribution of Tmax and HV after dichotomization at the median (Tmax 4.05, HV 28.5) is summarized in Table 4 (29 of 33 patients had non-missing data); these are labelled to indicate the expected outcome [Good (G), Bad (B)]. As expected, patients with Low Tmax and High HV (n = 9) had a shorter period of progression-free survival than those with High Tmax and Low HV (n = 14); P = 0.04 (Fig. 3E).

**Table 4:** Distribution of 33 patients by dichotomized Tmax and HV.

Tmax	HV		Total
	Low G	High B	
Tmax low B	5	9	14
Tmax high G	10	5	15
Total	15	14	29

After forward stepwise reduction, the final OS model contained Tmax, SUVmax, T1_Vol_nt, and nrCBV_et (Tmax HR = 0.11 P < 0.001, SUVmax HR = 2.14 P = 0.01, T1_Vol_nt HR = 2.07 P = 0.007, nrCBV HR = 1.55 P = 0.01). Restriction to Tmax and nrCBV_et revealed a significant decrease in time to death among those with nrCBV_et above the median and Tmax below the median (n = 8) relative to those with nrCBV_et below the median and Tmax above the median (p = 0.04) (Fig. 3F).

## Discussion

This is the first study, to our knowledge, to use multimodality imaging to explore tumor hypoxia, vasculature, and the correlation between radiographic features and outcomes in patients with Bev-refractory recurrent GBM.

The potential prognostic value of anatomic imaging of tumor volumes for OS and PFS in Bev-treated recurrent GBM remains controversial^25,26^. In this study, we sought to assess whether T1 weighted and FLAIR tumor volumes and their ratio are useful predictors of OS and PFS in these patients. Our results show that T1_Vol, T1_Vol_et, T1_Vol_nt and FLAIR_Vol are significantly associated with OS, but not PFS. The Vol_Ratio did not predict either OS or PFS.

Huang et al. assessed associations between tumor volumes and outcomes for recurrent GBM patients treated with Bev^27^; posttreatment enhancing volume and posttreatment FLAIR volume were significantly associated with OS and PFS. Ellingson et al. explored the relationship between conventional MRI tumor volume and survival for recurrent GBM patients treated with Bev^28^. Bev significantly reduced T1 weighted and FLAIR tumor volumes; both pretreatment and posttreatment FLAIR tumor volumes were not significantly correlated with OS and PFS. Further, both pretreatment and posttreatment T1 weighted enhancing tumor volumes were significantly correlated with PFS, but not OS. The pretreatment ratio of FLAIR to contrast enhancing tumor volume was a predictor of OS and PFS, unlike the posttreatment ratio of FLAIR to contrast enhancing tumor volume.

We calculated the ratio of FLAIR to contrast enhancing tumor volume; this ratio was not significantly correlated to OS and PFS in our cohort. We acquired data within 3 days prior to treatment. In contrast, Huang et al. acquired their data between 3 to 6 weeks after treatment while Ellingson et al. acquired their posttreatment data between 6 to 8 weeks after treatment. These inconsistencies may have affected the results^27^. As the anti-permeability effect of Bev may change over time, and thus potentially cause tumor volumes to also change with time, associations between tumor volumes and outcomes may vary accordingly. In another study, Schmainda et al. found that percent changes of rCBV relative to baseline at 2 and 16 weeks were significantly related to OS, but not at 8 weeks^16^. This suggests that correlations of OS and PFS to imaging biomarkers may be sensitive to when imaging is done.

In vivo tumor hypoxia and vasculature imaging could enhance our understanding of the pathophysiologic mechanisms of GBM, and in turn optimize timing and doses of radiotherapy and chemotherapy. Our results indicate that the median time to GBM progression increased significantly relative to historical data^29^; specifically, median PFS in the current study (53 days; 95% CI 42 to 113) was higher than historical controls (37.5 days; 95% CI, 34 to 42 days; P < 0.001). However, the median time to death in our study, 129 days (95% CI 86 to 199 days) or 4.3 months (95% CI 2.9 to 6.6 months), was not significantly different (P = 0.10) from the median time to death of 5.9 months (95% CI 4.4 to 7.6 months) more recently observed in 55 patients with recurrent GBM taking Bev beyond initial Bev progression^4^. PFS at 4 months in our study (31%) was not significantly different from PFS-4 (38%) in 99 patients who received subsequent therapy after progression on one of five consecutive, single-arm, phase II clinical trials evaluating bevacizumab regimens for recurrent GBM (P = 0.40).

Perfusion (srCBV) was tightly associated with hypoxia (HV, HVmean, TBpeak, TBmax, TB5percent), perhaps because most of the hypoxia and hypervascularization zones were in contrast enhancing areas^30^. Hypoxia (HV, HVmean, TBpeak, TBmax, TB5percent) and tumor volumes (T1_Vol, T1_Vol_et, Vol_Ratio) were also tightly associated. Whereas other PET and MRI parameters were weakly associated, as previously noted^24^, those results may be related to locating hypoxia zones in different ROIs^30^, or may indicate that these parameters have a unique and complementary role relative to tumor status^24^.

Normalized and standardized rCBV are the most common DSC-MRI metrics used for evaluating tumors^19^. We used spin echo (Dana Farber Cancer Institute) and gradient echo (UT Health at San Antonio) based methods to acquire our DSC data. The former is sensitive to capillary-sized vessels, whereas the latter is sensitive to a broad range of vessel sizes^19,31^. The value of the rCBV obtained with the gradient echo method is much larger than that obtained with the spin echo method in high-grade gliomas^32^. Some studies have confirmed that these two methods are comparable in terms of related parameters in in vivo studies^33,34^. However, srCBV has greater reproducibility^17^ than nrCBV and does not require the manual step of drawing a reference ROI, as does normalized rCBV. This may also explain why significant correlations were found with srCBV but not nrCBV. We also confirmed this finding with PCA, including all spin echo- and gradient echo-based DSC parameters. The mean within-subject difference for the first two principal components did not vary significantly with clinic site—or, therefore, with method (Fig. 2B).

Among DSC imaging metrics, rCBV is the most common for evaluating brain tumors^35^. Based on survival analysis, the mean values of srCBV (HR = 1.61, 95% CI 1.09 to 2.38, P = 0.02), srCBV_et (HR = 1.56, 95% CI 1.07 to 2.26, P = 0.02), and srCBV_nt (HR = 1.46, 95% CI 1 to 2.14, P = 0.05) were significantly associated with OS. Higher srCBV values were associated with poor outcome, which suggests that more vascular tumors convey a poorer prognosis. These results are consistent with several previous studies^14,36^. Patients with a within-subject mean nrCBV greater than 1.75 have a significantly shorter PFS than those with nrCBV less than 1.75^37^. We found that patients with a mean srCBV greater than 0.99 had a significantly shorter PFS than those with srCBV less than 0.99 (HR = 3.4 P = 0.02). This threshold is consistent with the tissue-validated thresholds previously determined by Hu et al^38^ and Prah et al^39^.

Tmax is considered a promising prognostic parameter^40^, although it is known to be complex, and may be affected by different factors, such as the arrival delay between the arterial input function and the tissue contrast agent concentration, arterial abnormalities that cause bolus temporal dispersion, and the MTT of the contrast agent, that mainly reflect the characteristics of microvascularization^40^. A reduction in Tmax may suggest high vascularity in a tumor^41^. We found that Tmax, Tmax_et, and Tmax_nt were significantly associated with OS and PFS, again suggesting that high vascularity in a tumor is associated with worse survival.

Hypoxia is exacerbated by antiangiogenic treatment and is important in tumor development, angiogenesis, and growth, and in treatment resistance. In our study, nrCBF and HV were positively correlated, suggesting that the larger the baseline HV value, the higher the CBF value in the tumor area after Bev treatment. A recent multicenter study of patients with newly diagnosed GBM reported a positive correlation between elevated nrCBF and HV^24^. Based on these data, we hypothesize that abnormal vascular anatomy and compromised vascular function within the tumor area contribute to heterogeneous blood flow and tumor hypoxia.

Evo is an investigational hypoxia-activated prodrug designed to be activated under hypoxic conditions in tumors. Evo plus Bev treatment in Bev-refractory GBM could therefore reduce HV and hypoxia-induced resistant tumors in Bev-refractory GBM^20^. In our study, HV was associated with worse OS and PFS. As shown in Table 3, higher HV reduced OS and PFS in the univariate proportional hazards regressions (P = 0.009 for PFS and P = 0.01 for OS). Spence et al. studied the HV and TBmax in newly diagnosed GBM before chemo- and radiation therapy with ^18^F-FMISO PET to assess their impact on OS and PFS; volume and intensity of hypoxia in GBM before radiotherapy were strongly associated with poor OS and PFS^42^. These results indicate that HV is a meaningful biomarker in GBM assessment and could provide more information than conventional anatomic imaging. In another study, Kawai et al. found a correlation between ^18^F-FMISO uptake in tumors and the expression of vascular endothelial growth factor, and confirmed that the volume and intensity of hypoxia were associated with OS^43^. Although their patients were newly diagnosed and ours were recurrent, these findings suggest that HV may be a reliable biomarker in tumor detection and treatment assessment in both settings. Additionally, based on our results, the timing of HV assessment does not change its reliability as an accurate marker.

Necrosis is an important characteristic of GBM tumors^44,45^ thought to be related to hypoxia^46,47^, and may affect treatment outcomes. Our data support a negative correlation between tumor necrosis and OS in our cohort of patients with recurrent GBM (Table 3), consistent with some studies of newly diagnosed GBM. Hammoud et al. reported negative correlations between degree of necrosis and OS^48^. Lacroix et al. found that smaller areas of necrosis were significantly associated with OS^49^. Taken together, these findings suggest necrosis might be an important biomarker.

We found that a negative change in HV was associated with longer OS and PFS and a positive change in HV was associated with shorter OS and PFS, although these results did not reach statistical significance. Yamaguchi et al. evaluated the performance of BEV treatment based on ^18^F-FMISO accumulation. They found that the ^18^F-FMISO responders had significantly longer OS than that of ^18^F-FMISO non-responders^50^. These results suggest that some patients with refractory GBM may benefit from combined Evo and Bev treatment and that HV may identify a subpopulation of Bev-refractory patients who would most benefit from this treatment. Further studies in larger populations are needed to confirm our results.

Our multivariate analysis results showed the final PFS model contained only Tmax and HV (Tmax HR = 0.54, p = 0.01, HV HR = 1.6, P = 0.046); Fig. 3E. For OS, Tmax, SUVmax, T1_Vol_nt and nrCBV_et (Tmax HR 0.11 P < 0.001, SUVmax HR 2.14 P = 0.01, T1_Vol_nt HR = 2.07 P = 0.007, nrCBV_et HR = 1.55 P = 0.01) remained in the model; Fig. 3F. These findings also require validation in a larger study.

There were some limitations to our study. Some variance in acquisition parameters and scanners were present at the two sites for perfusion data. Although the parameters calculated by these two methods are comparable in in vivo studies, there may be some differences not accounted for. We acquired our ^18^F-FMISO data at 2 h post ^18^F-FMISO injection. The time between ^18^F-FMISO injection and data acquisition may affect ^18^F-FMISO uptake^51^, with some studies suggesting ^18^F-FMISO data should be acquired at 4 h post ^18^F-FMISO injection^51^. Our study was also limited by the numbers of patients and time points. P-values are presented without correction for multiple testing, possibly increasing the Type 1 error, requiring interpretation considering correlation, biological plausibility, and consistency with other results in this study and with results published in other studies.

## Conclusions

In this cohort of 33 patients with bevacizumab-refractory GBM, hypoxia is a key biomarker for therapeutic efficacy. High hypoxia volume, enhancing and non-enhancing tumor volumes, and srCBV were all inversely correlated to patient outcomes. If validated in larger studies, these imaging biomarkers may be useful for detection of GBM, and for planning treatments and assessing responses.

## Supplementary Information

Supplementary Information.
