# Supplementary material for: Assessment of tumor hypoxia and perfusion in recurrent glioblastoma following bevacizumab failure using MRI and 18F-FMISO PET
Source: Sci Rep. 2021 Apr 7;11:7632. doi: 10.1038/s41598-021-84331-5 (PMC8027395; doi:10.1038/s41598-021-84331-5)
Supplement: Supplementary file 1 — Supplementary Information. [file 41598_2021_84331_MOESM1_ESM.docx]

**Assessment of tumor hypoxia and perfusion in recurrent glioblastoma following bevacizumab failure using MRI and ^18^F-FMISO PET**

Author’s Full Names: Shiliang Huang^1^, Joel E Michalek^1^, David A. Reardon^2^, Patrick Y. Wen^2^, John R. Floyd^1^, Peter T. Fox^1^, Geoffrey D. Clarke^1^, Paul A. Jerabek^1^, Kathleen M. Schmainda^3^, Mark Muzi^4^, Hyewon Hyun^5^, Eudocia Quant Lee^2^, Andrew J. Brenner^1,6^

Affiliation: ¹University of Texas Health San Antonio Cancer Center (A.J.B.), The University of Texas Health Science Center at San Antonio, 7703 Floyd Curl Drive, San Antonio, Texas 78229-3900

^2^Dana-Farber Cancer Institute, Boston, MA

^3^Departments of Radiology and Biophysics, Medical College of Wisconsin, Wauwatosa, WI.

^4^Department of Radiology, University of Washington, Seattle, Washington

^5^ Division of Nuclear Medicine, Department of Radiology, Brigham and Women’s Hospital, Boston, MA

^6^Corresponding Author: Andrew J. Brenner, Telephone: 210-450-5936, Fax: 210-692-7502, Email Address: dr_brenner@yahoo.com

**Supplementary Data: Glossary**

**GLOSSARY**

^18^F-FMISO PET: 18F-Fluoromisonidazole positron emission tomography

ADC: Apparent diffusion coefficient. The unit is 10^-6^ mm^2^/s

Bev: Bevacizumab

CBF: Cerebral blood flow. The unit is ml/min/100g of tissue

CBV: Cerebral blood volume

CI: confidence interval

DCE-MRI: Dynamic contrast-enhanced MRI

DSC-MRI: Dynamic susceptibility contrast MRI

DWI: Diffusion weighted MRI

ECOG: Eastern Cooperative Oncology Group

Evo: Evofosfamide

FLAIR: Fluid attenuated inversion recovery

FLAIR_Vol: The tumor volume (cm^3^) on the FLAIR MRI image

FLAIR∆T1: FLAIR_Vol excluded T1_Vol, it is a region of brain edema and invasion.

GBM: Glioblastoma multiforme

HV: Hypoxic volume (cm^3^)

HVmean: The mean of the pixel intensity within HV

HR: Hazard ratio

MRI: Magnetic resonance imaging

MTT: Mean transit time. The unit is second.

MTT, TTP, Tmax, nrCBV, rCBF, srCBV, ADC: Calculated in the area covered by T1_Vol ROIs

MTT_et, TTP_et, Tmax_et, nrCBV_et, rCBF_et, srCBV_et, ADC_et: Calculated in the area covered by T1_Vol_et ROIs

MTT_nt, TTP_nt, Tmax_nt, nrCBV_nt, rCBF_nt, srCBV_nt, ADC_nt: Calculated in the area covered by T1_Vol_nt ROIs

nrCBF: Normalized relative cerebral blood flow. There is no specific unit for this normalized relative parameter

nrCBV: normalized relative cerebral blood volum. There is no specific unit for this normalized relative parameter

OS: Overall survival

PCA: Principal component analysis

PET: Positron emission tomography

PFS: Progress-free survival

RANO: Response Assessment in Neuro-Oncology

rCBF: Relative cerebral blood flow. There is no specific unit for this relative parameter

ROI: Region of interest

srCBV: Standardized relative cerebral blood volum. There is no specific unit for this standardized relative parameter

SUV: Standardized uptake value

SUVmax: The maximum standardized uptake value

SUVpeak: The average SUV within a circular 1cm diameter region of interest (ROI) centered on a high-uptake part of the tumor

T1/FLAIR: The ratio of the T1_Vol/ FLAIR_Vol

T1_Vol: The tumor volume (cm^3^) on the T1 weighted MRI image

T1_Vol_et: The enhanced tumor volume (cm^3^) on the T1 weighted MRI image

T1_Vol_nt: The necrotic tumor volume (cm^3^) on the T1 weighted MRI image

T1_Vol= T1_Vol_et+ T1_Vol_nt

TB: The tissue to cerebellum ratio image. In other words, the PET image normalized by the mean of the ROI on the cerebellum area

TB5percent: The average of the top 5% TBs within T1_Vol

TBmax: The maximum intensity value of the tissue to cerebellum ratio image within T1_Vol

TBpeak: The average of the top 200 TBs (TBs: the pixels of the tissue to cerebellum ratio image) within T1_Vol

Tmax: Time-to-maximum of the residue function. The unit is seconds

TTP: Time to peak. The unit is seconds
